# Peer Review for “Checklist Approach to Developing and Implementing AI in Clinical Settings: Instrument Development Study”

**DOI:** 10.2196/69593

**Published:** 2025-02-20

**Authors:** Keith Thompson

**Affiliations:** 1 Department of Family Medicine, Western University, 1151 Richmond St, London, Canada, 1 519-661-2111

**Keywords:** artificial intelligence, machine learning, algorithm, model, analytics, AI deployment, human-AI interaction, AI integration, checklist, clinical workflow, clinical setting, literature review

*This is the peer-review report for “Checklist Approach to Developing and Implementing AI in Clinical Settings: Instrument Development Study.”*

## Round 1 Review

### General Comments

 This paper [1]...is a very cohesive approach to establishing a framework for the implementation of artificial intelligence (AI).

### Specific Comments

#### Major Comments

1. Ideally there should be information on the demographics of the expert panel.

2. Please add comments regarding equitable access for these technologies.

## Abbreviations

AI: artificial intelligence
